# Factors affecting dental service utilisation among older Singaporeans eligible for subsidized dental care – a qualitative study

**DOI:** 10.1186/s12889-019-7422-9

**Published:** 2019-08-08

**Authors:** Rakhi Mittal, Mun Loke Wong, Gerald Choon-Huat Koh, Desmond Luan Seng Ong, Yun Hui Lee, Mei Na Tan, Patrick Finbarr Allen

**Affiliations:** 1 Faculty of Dentistry, National University of Singapore, Singapore, Singapore; 2 Saw Swee Hock School of Public Health, National University of Singapore, National University Health System, Singapore, Singapore; 3 National University polyclinic, Jurong, Singapore

**Keywords:** Elderly, Dental care subsidies, Older population, Dental care utilisation

## Abstract

**Background:**

The World Health Organization has highlighted the paucity of research into the oral health needs of older adults. In Singapore, until recently, publically funded/subsidized oral health care for adults has been limited to basic primary care at government-funded polyclinics. Access to a more comprehensive range of subsidized care in the private sector was widened through the government-funded Community Health Assistance Scheme (CHAS) in 2012 and Pioneer Generation (PG) scheme in 2015. Little is known about the attitude to dental service utilization among older adults in Singapore since then.

**Methods:**

We conducted semi-structured individual interviews with 25 participants above 65 years of age who were eligible for subsidized dental care plans. Participants were recruited from a public teaching hospital and a public primary care clinic in Singapore. The duration of each interview was 15–30 min. Interviews were transcribed verbatim and the transcripts were analyzed thematically using a phenomenological approach.

**Results:**

Pertinent themes emerged related to four major areas: (a) general awareness towards oral health, (b) life course perspective of oral health, (c) barriers to visit the dentist, (d) shaping dental service utilisation behaviours through provision of financial subsidies for dental care. Most participants perceived a strong relationship between oral health and systemic health. However, there were erroneous traditional beliefs such as oral health is not part of physical health and edentulous participants did not need to visit a dentist. Fear, anxiety, previous negative experience and lack of knowledge were barriers to visiting the dentist. Trust and convenience were considerations for patients when deciding whether to switch from public to private dental services where CHAS/PG were only available.

**Conclusion:**

Our study provided important insights regarding oral health perceptions and beliefs of older people residing in the community which may affect their dental service utilization. This further highlights the importance of understanding the concerns of this group when implementing healthcare policies for elderly in Singapore. The findings of our study will serve as a baseline for future studies in Singapore and inform studies in other countries that implement targeted schemes for older adults.

## Background

An inevitable consequence of major demographic shifts caused by ageing and lower birth rates has been the evolution in the age structure of the world’s population [1]. The number of persons aged 60 years or older in the world was estimated to be 605 million in 2000 which is projected to grow to nearly 2 billion by 2050 [2]. In most respects, population ageing is a testimony of the great success in healthcare. In coming years, policy makers will face challenges posed by chronic diseases in old age. Interestingly, chronic diseases and most oral diseases share common risk factors [3]. Oral health of the elderly population is a global concern which involves a high prevalence of tooth loss, dental caries, periodontal disease, hypo-salivation and oral cancers. According to the World Health Organization (WHO), oral health is an integral part of general health and an important determinant of quality of life (QoL) [4]. This indicates that oral health policies should be a part of national healthcare strategies.

It has been shown that dental care utilisation is low among older people, particularly among the socio-economically disadvantaged due to significant barriers [5]. Previous literature suggests that there is no single factor that stands out as being most significant barrier to access oral health care among older population. Dental care cost, shortage of professionals, and lack of awareness with regards to services provided and location of facilities have been important barriers to the utilisation of dental services among older adults [6, 7]. Beside dental care cost, oral health literacy, lack of perceived need for care, disability and dental fear are also important factors influencing dental visits by the elderly population. For example, in Japan, under the universal health insurance system established in 1961, people living in Japan are eligible to have medical care including dental care, at a subsidized price (10–30% of the total cost of treatment) [8]. One of the cross-sectional studies done to analyse the oral health status of homebound elderly in Japan revealed that utilization of dental care is limited due to the lack of perceived need for care, issues related to transportation and fear [9]. Interestingly, economic status is a major predictor affecting oral health in Japan where universal health insurance covers most dental care expenses [10]. Another barrier which is frequently cited in the literature relates to the shortage of manpower and low priority allocated to oral health by national health authorities [3].

Singapore’s healthcare system is founded on three essential elements, namely: [1] sufficient range of health care services, [2] appropriate quality, and [3] affordability [11]. In 2000, the Ministry of Health (MOH) started the Primary Care Partnership Scheme (PCPS) to ensure that low-income elderly Singaporeans could receive subsidised care for common acute illnesses at General Practitioner (GP) clinics near their homes. The scheme was targeted at those 65 years and above with a per capita household monthly income of no more than $700 per person. In 2002, selected dental conditions were also included under PCPS [12]. In 2012, PCPS was re-named Community Health Assist Scheme (CHAS) and continued to be significantly enhanced over the years since then. In 2014, the age criterion was removed and the eligibility threshold based on the annual value of patient’s residence was increased from $13,000 to $21,000 [12]. This enabled about half of all Singaporean households to benefit from the scheme [12]. With the inclusion of selected dental conditions in CHAS, subsidised oral health care for adults has been extended beyond government-funded polyclinics to the private sector through the government-funded CHAS programme. The Pioneer Generation (PG) scheme was launched in 2015, to honour and recognise pioneers (special generation of Singaporeans who were at least aged 16 years in 1965 or are aged ≥65 years by the end of 2014) for their hard work and dedication. Under this scheme, all Singaporean citizens over 65 years of age receive additional subsidies on top of existing services and medication at polyclinics, specialist outpatient clinics, general practitioners and dental clinics. Almost 97% of CHAS and PG cardholders island-wide have access to more than one CHAS clinic within a kilometre of their homes [13].

While the introduction of CHAS and PG initiatives was intended to encourage utilisation of private primary care, patients regarded them as nothing more than financial assistance [14]. Rapid changes to the national healthcare financing landscape may create confusion among the vulnerable population which results in a lack of understanding about the intentions behind such policies. If policy goals are not properly communicated to the target population, it could result in a lack of public awareness which could in turn lead to a failure of these initiatives [15]. It has been widely accepted that subsidised dental care or dental insurance programmes should be assessed based on two criteria [16]. The first criteria is related to how effective the programme is in *treating* dental disease and the second is how effective the programme is in *preventing* dental disease. To date, there has been no study investigating the role of oral health subsidies (under CHAS and PG card) on dental care utilisation among the elderly. The aim of the present study is to understand the factors affecting dental care utilisation among older Singaporeans who were eligible for CHAS or PG subsidies.

## Methods

### Study design

We conducted a descriptive qualitative study among older Singaporeans. We used purposive sampling, participants were approached while waiting to see their dentist/medical practitioner in two locations: Faculty of Dentistry (FoD) Clinic, National University of Singapore (NUS) and National University Polyclinic (NUP), Jurong. Faculty of Dentistry is located within National University of Singapore (NUS). Since FoD setting is very specific to dental care whereas polyclinic setting is community based, hence selection of two research sites gave us the opportunity to recruit a spectrum of older adults with differing dental experiences and attitudes.

### Study setting

A semi-structured interview guide was developed through consultation with experts in geriatric dentistry and public health (Table 1). The interview questionnaire focussed on areas such as [1] general awareness about oral health, [2] change in attitude towards oral health during old age, [3] barriers to visit the dental clinic, and [4] knowledge about dental care subsidies and their utilisation. The interview guide was pilot tested followed by minor amendments. It only served to prompt the interviewer to cover potential areas as interviewees were allowed to freely lead the discussion, allowing the emergence of novel topics and ideas which may not have been included in the guide. The interviewer had no prior relationship with the participants. The interviews were audio-recorded with the participant’s consent and transcribed verbatim. Field notes were taken down at the time of interviews and reflexivity was used during data analysis.

**Table 1:** Semi-structured interview guide

Main questions	Probing questions
1) Influence of oral health on general health	a) How the state of your mouth affects the way you feel in yourself? b) How has the state of your mouth impacted your physical health (e.g. digestion or bowels)? c) In what ways do you believe that the condition of your mouth has an impact on your general health?
2) Oral health in the past and now	a) When you were young, did your parents bring you to the dentist for regular check-ups or only when needed? b) [If subject said that he/she did not see a dentist regularly in the past but see a dentist regularly now (or vice versa)]: Why so? Did anything happen to cause this change(s) in attitude(s)?
3) Past dental care experiences and perceived barriers	a) How easy was it for you to get dental treatment? b) How often do you see your dentist now? Do you go for regular check-ups or only when you have problems with your teeth? c) To what extent do the costs of dental treatment influence you going to the dentists? d) Have you ever had a particularly bad experience with a dentist? What happened? Did it put you off from going to a dentist?
4) Access to dental care subsidies and their utilization	a) Are you aware of the possibility of dental care through CHAS or PG card? b) How easy is it for you to get dental treatment using a CHAS or PG card? c) How far is the dental practice from your home/work? How do you usually travel to the dental practice when you go to see your dentist? Does the distance you have to travel influence your decision?

### Data collection

Twenty-five individual in-depth interviews were conducted by the main author (RM) between October 2017 and May 2018. We tried to ensure a varied range of subjects in our study population: e.g. dentate, partially edentulous (e.g. wearing removable partial dentures) and completely edentulous. Participant recruitment criteria included: (i) aged 65 years old and above, (ii) eligible for subsidised dental care plans including either CHAS or PG subsidy OR both, and (iii) able to understand English, Chinese or Malay. The identity of participants was kept confidential and written consent was obtained prior to interviews. Each interview took between 15 and 30 min and were conducted in the waiting area of both research sites according to the convenience of participants.

All participants were physically mobile. For non-English speaking participants, translators helped interviewer to translate English to Chinese/Malay and also Chinese/Malay to English. The objectives of the study were clearly explained to each participant and they were assured of the anonymity and confidentiality of their responses. It was explained that their participation was voluntary and they could withdraw from the study at any time. Ethics approval was obtained from the National health Group Domain Specific Review Board (DSRB Reference Number: 2017/00223). There was one repeat interview where a participant was called again to complete the remaining questions.

### Data analysis

In this study, each interview was transcribed and studied several times using a phenomenological approach in order to extract primary codes. The author and co-authors were trained in qualitative research methods and themes/subthemes were identified and validated after multiple discussions, with a focus on identifying and describing both implicit and explicit ideas within the data [17].

Following the transcription of the first few interviews, a process was used by research team members to confirm persistent themes. We were responsive and open to what was in the data and allowed the data to guide our iterative approach to analysis. Study team members including (RM, PFA, WML) reviewed emerging findings during monthly team meetings until data saturation (recruitment of additional cases no longer provides additional information or insights) occurred and used consensus among study team members to resolve disagreements.

### Rigor/trustworthiness

The criteria of credibility, dependability and conformability were used to confirm the rigor of study findings. Following the transcription of the first few interviews, a peer review process was used by research team members to confirm persistent themes. We adhered to all assumptions and strategies of the qualitatively driven designs. We were responsive and open to what was in the data and allowed the data to guide our iterative approach to analysis. We reviewed emerging findings during monthly team meetings to ensure data saturation and consensus among study team members. This was done to ensure the credibility of research.

## Results

Twenty-five individual in-depth interviews were conducted by the main author (RM). Participants’ characteristics are described in Table 2. All participants were familiar with CHAS and PG card system. Major consistent themes affecting dental care utilisation which emerged included: general awareness towards oral health, life-course perspective of oral health, barriers to visit the dentist and shaping dental service utilisation behaviours through dental subsidies (Table 3).

1. General awareness about oral health

- Awareness of relationship of oral health with general well being

**Table 2:** Characteristics of study participants

Variables	N (%)
Age	
65 years – 70 years	11 (44)
>70 years	14 (56)
Gender	
Male	10 (40)
Female	15 (60)
Dentures	
Fully dentate	6 (24)
Partially edentulous	
With denture	14 (56)
Without denture	2 (8)
Completely edentulous (with or without denture)	3 (12)

**Table 3:** Themes related to factors affecting dental service utilization among older Singaporeans using subsidized dental care

Themes	Sub - themes
1) General awareness about oral health	• Awareness of relationship of oral health with general well being
2) Life-course perspective of oral health	• More positive attitudes towards oral health from childhood to old age
3) Barriers to visit dental clinic	• Fear, anxiety and past negative experience with dentist • Do not perceive need to visit dentist • Lack of awareness
4) Shaping dental service utilisation behaviours through dental subsidies	• Effect on frequency of dental visits, before and after CHAS/PG • Effect on shift from public to private dental clinic after CHAS/PG

*CHAS* Community Health Assistance Scheme

*PG* Pioneer Generation

Most participants perceived a strong relationship between oral health and systemic health. They were able to explain the role of teeth during eating and chewing which can eventually affect their physical health.

*P1: “I think oral health affects our body because if we cannot chew food properly … cannot bite, just swallow, then the body cannot be strong.”*

*P2:* “*… if we eat and don’t chew properly, then we will get indigestion etc.”*

Majority of the responses reflected that the elderly predominantly associated the word ‘teeth’ with ‘eating and chewing’. One participant mentioned that “bad oral health” may lead to serious heart disease because of earlier work experience in the dental clinic.

*P7: “So far I know our heart attack all comes from the mouth. If the mouth is dirty then all get affected … Last time, my boss was a dentist.”*

Few participants were unaware of a relationship between oral health and systemic health due to lack of knowledge or a traditional belief that oral health is not part of physical health.

*P4: “I don’t know whether it can affect or no … maybe I have not gone through the experience. The only thing I know is when your teeth are bad, you get a toothache.”*

*P11: “… I believe there is no connection between mouth and body. Both are separate.”*

2. Life course perspective of oral health

- *More positive attitudes towards oral health from childhood to old age*

It was found from the interviews that the participants’ attitudes and perceptions changed slowly due to increase in tooth-related problems from childhood to old age. It was reflected from the interviews, to avoid tooth pain and teeth related problems, participants started their routine visits. This change in attitude increased their dental care utilisation during old age. When asked about their present frequency of dental visits, 40% of participants had twice or more a year, 20% responded once a year, 24% said only when required and 16% never visited dentists.

*P9: “I found it’s not easy to eat with false teeth … , I didn’t take care of my teeth when I was young, they got decayed and doctor pulled until no more teeth. Now, I (have) difficulty in eating.”*

*P11:* “*… because of my age, I started monitoring my dental appointments regularly.”*

*P18: “Now I want to save teeth because my back teeth are missing which makes me difficult to eat.”*

3. Barriers to visiting the dental clinic

- *Fear, anxiety and past negative experience with dentist*
  Although most of the participants agreed that oral health was important, they remained reluctant to visit a dentist because of fear of physical pain and discomfort. It was apparent that past dental experiences made strong and long-lasting impressions on the minds of the elderly. Participants who had a bad experience due to pain or side effects of medicines after extraction or anaesthesia, tried to avoid subsequent dental visits.

*P3: “I had a bad experience with a dentist. He just pulled my tooth … I saw my tooth had no problem … he gave me antibiotics. After that, I had diarrhoea.”*

*P9: “I am always worried because I am very scared of tooth polishing which I had last time.”*

*P10: “I am scared … Last time, injection didn’t work, (but) dentist still pulled my teeth.”*

b) *Do not perceive the need to visit a dentist without teeth*
  Presence of teeth was also one of the reasons affecting dental care utilisation. It was highlighted that edentulous participants did not feel the need to visit a dentist.
*P2: “Now I have no teeth, so no need to visit a dentist.”*
c) *Lack of awareness*
  It was found that few participants believed that tooth extraction was the only option, not realising that “tooth repair” was an alternative option. Underlying cultural beliefs and practices influenced dental care seeking behaviours and potentially the condition of their teeth and mouth.
*P4: “I don’t like to go to the dentist, its horror, and nightmare … when I have pain in tooth, then the only option left for me is extraction.”*
*P14: “I am afraid that if I visit a dentist to repair my teeth, the filling will come out again.”*

4. Shaping dental service utilisation behaviours through dental subsidies

- *Effect on the frequency of dental visits, before and after CHAS and PG subsidies*

It was highlighted that participants who had regular dental visits prior to the introduction of the CHAS or PG benefits were also more likely to maintain their regular dental attendance or visit the dentist more often.

*P16: “My friend told me about this blue CHAS card … now I visit twice a year for a dental wash.”*

*P17: “Without PG card it was occasionally when required. With this card, I visit more often until I reach limit because they kept a certain amount.”*

*P18: “With this card, I started going for a routine check-ups. But earlier, I used to visit (only) when I had pain.”*

*P23: “Earlier it was once a year, now (I) can visit 3-4 times a year.”*

A few participants highlighted that their frequency of dental visits remained unchanged even after receiving subsidized dental care.

*P19: “Earlier … I used to go one-year-one-time and now, I (still) go one-year-one-time to a dentist.”*

*P20: “I don’t want to go to the dentist much. If any problem, then I do …*.”

*P21: “ …*. *it’s 2 times free a year but (I) still never went to a dentist for washing.”*

*P22: “I have never used PG card and CHAS blue card. I use my Medisave account because Government adds $200 every year in (my) Medisave account.”*

b) *Effect on shift from public to a private dental clinic after CHAS/PG*.

When participants were asked about their preference to visit a public (polyclinic) or private dental clinic with access to subsidised dental care plans, mixed opinions surfaced during the interviews. Some participants considered their trust towards dentist as a vital component because of fear and anxiety associated with the treatment.

*P11: “I follow the same doctor from last 10 years and I still follow him … for us, we still follow Government doctor. When we really can’t take an appointment, then only we go to private.”*

*P13: “My present dentist is very friendly, so before the card, I was going to the same private dentist.”*

*P15: “I visit the same private dentist because can get an appointment very fast.”*

*P24: “I heard polyclinic is better, so I always come to polyclinic.”*

*P25: “Polyclinic is very near to my home and I come here for my medical appointments (as well) so it’s convenient for me.”*

With the introduction of CHAS/PG initiatives, some participants reported that they switched to the private sector instead of the public sector for their oral health care needs. A few participants also switched to private care due to long waiting times in the public sector.

*P16: “Earlier I was going to the government clinic. Now I go to private.”*

One of the participants showed dissatisfaction and felt that his private dental professional failed to treat his dental problems. For this reason, he had to come back to polyclinic for his dental visits.

*P23: “I went to the private clinic but (was) not satisfied. After that, I started coming to polyclinic.”*

Hence, factors including trust towards dentist and convenience were cited as equally important considerations for the elderly when they were deciding on whether to access public or private dental services. Cost was not the only consideration for many of them.

## Discussion

Like many other developed countries, Singapore has undergone a major demographic and epidemiological transition in recent decades, especially with a rapidly ageing population. Little was known about the oral health knowledge and dental care utilization of elderly Singaporeans. Therefore, the need for greater insight into the relationship between their perception, attitude and dental service utilization is essential. Our study is first of its kind to show dental care utilization behaviour among older Singaporeans after the introduction of public oral health funding. Literature reports that public funding for dental care may not result in equitable utilisation, and may lead to greater socioeconomic inequalities [18]. It may be because well-educated and higher socio-economic status patients will be more likely to avail this funding because of greater knowledge/awareness of post-treatment benefits [19]. Dental care utilization among the elderly population is vulnerable to inequalities based on their poorer knowledge and reduced post-retirement income resources. It has been shown that the longer life expectancy is not always accompanied by better quality of life or optimal conditions of oral health [20]. To our knowledge, this qualitative study is first of its kind to explore the factors affecting dental care utilisation among older Singaporeans who are eligible for subsidised dental treatment. Four major themes emerged from the interviews: [1] general awareness towards oral health, [2] life-course perspective of oral health, [3] barriers to visit the dental clinic, [4] shaping dental service utilisation behaviours through dental subsidies.

### General awareness towards oral health

In our study, oral health was perceived as an important aspect of general health among the participants. Interestingly, majority of participants explained this relationship because of the need for natural teeth to chew food. The Chinese population has historically been found to have a strong belief in the relationship between diet and health [21]. It was not surprising that the oral-systemic relationship was not well appreciated by majority of the participants. It was found that majority of participants held positive attitudes toward their current oral health status and were aware that proper oral care is a pre-requisite to good oral health.

### Life-course perspective of oral health

According to the findings of our study, participants became more aware of their oral health and were keen to save natural teeth during old age. During the time of interview, majority of the participants had dental visits twice or more a year. It was reported that for many Chinese, extraction was the only remedy to a prolonged uncontrollable toothache and loss of one or even a few teeth was not considered a problem [22]. During the mid-1900s, due to a lack of knowledge among patients and a lack of skills and resources among dental professionals, extraction was the simplest solution irrespective of the age of a patient [23]. Interestingly, the prevalence of edentulism among elders above 65 years of age was higher in Singapore (21%) compared to China (11%) in 2005 [3].

### Barriers to visit the dental clinic

It has been widely accepted that dental care utilisation is low among older people, particularly among the socio-economically disadvantaged population [3]. Potential barriers which were highlighted during the interviews included [1] fear, anxiety or past negative experience with a dentist, [2] unable to perceive the need to visit dentist, and [3] lack of awareness. Dental fear or anxiety is generally underestimated in older persons [24] as it is difficult to draw a line between ‘lack of perceived need’ and ‘dental fear’. Often, the ‘need’ for dental care is associated with the presence of natural teeth. For example, in our study, edentulous participants indicated ‘no need’ to visit a dentist. It takes time to change the beliefs and behaviours of many older people. Dental professionals are considered to be an important source of oral health education but this is limited to individuals who actively seek dental care services [25]. Good oral health knowledge may help to prevent potentially debilitating oral conditions that can reduce the ability to properly masticate which affects nutrition in old age [26].

Elders tend to avoid dental visits due to numerous factors which results in decreased dental care utilization [27]. They find it difficult to access dental care facilities in public sector due to shortage of resources. It is not practical to have equal number of professionals to take care of seniors in the community with increased ageing population. Task shifting is one of the options discussed by the WHO [28] to strengthen the workforce to rapidly increase access to health care resources, and has been implemented in several countries [29]. At community level, allied health care workers should be involved to educate/take care of seniors. Local government should integrate foreign domestic workers/caregivers into the formal network, which may minimize the load of dental professionals in the community. Knowledge about the importance of oral health among elderly and policy makers is a key driving factor for future change. Though the reasons for non-utilization of dental care services are multidimensional, it may differ with personal context and attitude [30]. Dental check-up of older population should be included as part of their general health care routine. This may help to widen the knowledge related to ‘need’ of routine dental care visits.

### Shaping dental service utilisation behaviours through dental subsidies

Majority of study participants were aware of subsidised dental care plans available for older Singaporeans. We found that elders who had better oral health knowledge and were visiting the dentist at least once in 2 years, started routine dental visits; whereas those who were visiting the dentist when they had a problem still followed the same habits, irrespective of the availability of subsidies. A study done to evaluate the effect of a policy to expand the Korean National Health Insurance benefit coverage to include dental scaling at the national level showed that prevalence of unmet dental care needs decreased by 6.1% and the likelihood of preventive dental care utilisation increased by 14.0%. Further sub-group analysis revealed that policy effect was limited only to high-income households [31]. These findings clearly reflect the ‘inverse care law’, where a person with high income uses services more compared to people with low income who are in relatively poor health. Also, participants who were keen to save their natural teeth had regular dental visits compared to those who considered ‘old age’ as a major factor for poor oral health.

The importance of trust in relationship with the dentist was reflected during our interviews. CHAS subsidies are only applicable in the private sector whereas PG subsidies are available both in public and private sector. Usually in polyclinics, there is a long waiting time. However, patients continue to follow the same dentist in the polyclinics. This reinforces the need for patients to have a trusting relationship with their dental professionals. Concerns regarding financial issues and inconvenience of accessing care can be alleviated when a relationship is strong [25].

The main limitation of our study is that participants were recruited from one geographical location of Singapore so our findings may not be representative of the entire elderly Singaporeans. The major strength of our study is that it is the first to document potential drivers of dental care utilisation among elders after the implementation of oral health subsidies. Each country has varied healthcare policy system and needs of older adults are heterogeneous, hence it is not easy to compare our findings with other countries. However, we believe the findings of our study will serve as a baseline for future studies in Singapore and inform studies in other countries that implement targeted schemes for older adults.

## Conclusion

The findings of our present study indicate that elders based their current oral health knowledge and dental care utilisation on experiences and information from many decades ago, highlighting the importance of a life-course approach towards oral health. They need adequate information about the resources available in the community for their efficient utilisation. Decision makers should design effective and affordable oral health strategies integrated into general health programmes for better oral health of the elders.

## Data Availability

The transcripts/data of this qualitative study are not publicly available due to confidentiality agreements with the participants.

## Abbreviations

CHAS: Community health assist scheme
GP: General practitioner
MOH: Ministry of health
NUP: National University Polyclinic
NUS: National University of Singapore
PCPS: Primary care partnership scheme
PG: Pioneer generation
QoL: Quality of life
